# The Role of Circulating Biomarkers in the Oncological Management of Metastatic Renal Cell Carcinoma: Where Do We Stand Now?

**DOI:** 10.3390/biomedicines10010090

**Published:** 2021-12-31

**Authors:** Alessandra Cinque, Anna Capasso, Riccardo Vago, Michael W Lee, Matteo Floris, Francesco Trevisani

**Affiliations:** 1 Biorek S.r.l., San Raffaele Scientific Institute, 20132 Milano, Italy; alessandra.cinque@biorek.eu; 2 Department of Medical Oncology Livestrong Cancer Institutes, Dell Medical School, University of Texas at Austin, Austin, TX 78712, USA; anna.capasso@austin.utexas.edu; 3 Urological Research Institute, San Raffaele Scientific Institute, 20132 Milano, Italy; vago.riccardo@hsr.it; 4 Faculty of Medicine and Surgery, Università Vita-Salute San Raffaele, 20132 Milano, Italy; 5 Department of Medical Oncology and Medical Education, Livestrong Cancer Institutes, Dell Medical School, University of Texas at Austin, Austin, TX 78712, USA; Lee_Michael@austin.utexas.edu; 6 Nephrology, Dialysis, and Transplantation, Università degli Studi di Cagliari, G. Brotzu Hospital, 09134 Cagliari, Italy; matt.floris@gmail.com; 7 Unit of Urology, San Raffaele Scientific Institute, 20132 Milano, Italy

**Keywords:** liquid biopsy, renal cell carcinoma, target therapies, biomarkers, circulating tumor cells, circulating tumor DNA, noncoding RNA, circulating proteins, metastatic renal cell carcinoma

## Abstract

Renal cell carcinoma (RCC) is an increasingly common malignancy that can progress to metastatic renal cell carcinoma (mRCC) in approximately one-third of RCC patients. The 5-year survival rate for mRCC is abysmally low, and, at the present time, there are sparingly few if any effective treatments. Current surgical and pharmacological treatments can have a long-lasting impact on renal function, as well. Thus, there is a compelling unmet need to discover novel biomarkers and surveillance methods to improve patient outcomes with more targeted therapies earlier in the course of the disease. Circulating biomarkers, such as circulating tumor DNA, noncoding RNA, proteins, extracellular vesicles, or cancer cells themselves potentially represent a minimally invasive tool to fill this gap and accelerate both diagnosis and treatment. Here, we discuss the clinical relevance of different circulating biomarkers in metastatic renal cell carcinoma by clarifying their potential role as novel biomarkers of response or resistance to treatments but also by guiding clinicians in novel therapeutic approaches.

## 1. Introduction

Renal cell carcinoma (RCC) represents one of the deadliest tumors worldwide, accounting for 3% of all malignancies [1]. Among the genitourinary neoplasms, RCC is the one with the highest mortality, with approximately a 76% overall survival rate [2]. The global incidence and mortality of RCC has increased in the last decade, with over 400,000 new cases diagnosed annually and 140,000 deaths [3]. However, the rate of mortality varies among countries due to the disparities in medical settings, such as the availability of frequent imaging and effective systemic oncological treatments. [4]. Therefore, there is imperative to discover reliable biomarkers that can be used for early diagnosis and accurate methods to detect RCC in patients. Circulating biomarkers represent an attractive platform of diagnosis and longitudinal monitoring and could have a prognostic and predictive role across several urological malignancies. Their role has been recently reviewed in bladder cancer [5,6,7] and prostate cancer [8,9]. The scope of this review is to discuss the clinical relevance of circulating biomarkers in the oncological management of metastatic renal cell carcinoma patients.

## 2. Histologic Classification of RCC

According to the World Health Organization, there are three major histological subtypes of RCC, all differentiated by histological and molecular genetic changes: clear cell (70%), papillary (10–15%), and chromophobe (4–5%) [10]. Both clear cell (ccRCC) and papillary (pRCC) cancers originate from the renal proximal tubule, while chromophobe (chRCC) arises from the distal part of the nephron [11]. Each type of histology displays diverse morphology but also different genetics and behavior. The remaining 10% of renal tumors include a variety of uncommon, sporadic, familial carcinomas and a group of unclassified carcinomas [12]. Briefly, minor histological subtypes are: oncocytoma, angiomyolipoma, collecting-duct carcinoma, sarcomatoid RCC, unclassified RCC, multilocular cystic RCC, papillary adenoma, renal medullary carcinoma, translocation carcinoma, mucinous tubular and spindle cell carcinoma, metanephric adenoma, adenofibroma, metanephric stromal tumor, renal epithelial and stromal tumors, and hereditary kidney tumors (Von Hippel–Lindau Syndrome, hereditary pRCC, Birt–Hogg–Dubé Syndrome, hereditary leiomyomatosis, tuberous sclerosis, and constitutional chromosome 3 translocation) [11,13,14,15,16].

## 3. Risk Factors and Genetics of RCC

RCC shows a 1:5:1 male predominance, peak incidence between 60 and 70 years, with a median age at diagnosis of 64 years of age [17]. The main risk factors responsible for the development of RCC include tobacco smoke, obesity, hypertension, diabetes, chronic kidney disease, exposure to toxic compounds, abuse of analgesics, genetic predispositions, and hereditary syndromes [18]. A number of different gene mutations have also been identified that suggest the potential for genetic predisposition. For example, investigation into familial RCC has highlighted different mutations in 11 genes (BAP1, FLCN, FH, MET, PTEN, SDHB, SDHC, SDHD, TSC1, TSC2, and VHL), some of which were also related to sporadic RCC onset. Von Hippel–Lindau (VHL) genes have been well elucidated as one of the main actors in carcinogenesis [19]. This is particularly evident in ccRCC, which is characterized by a highly vascularized morphological structure, where the VHL tumor suppressor gene is often inactivated. As a result, the subsequent over-expression of the hypoxia-inducible factor (HIF)-2alfa oncoprotein promotes a direct effect on its downstream targets including the vascular endothelial growth factor (VEGF) [20].

## 4. Diagnosis

The diagnosis of RCC remains cumbersome in clinical practice. In fact, the most common RCC clinical features, such as gross hematuria, flank pain, palpable abdominal mass, and fever are observed in a minority of patients, whereas the majority display nonspecific symptoms that can be easily mistaken for other conditions or distinct types of cancer [21]. For this reason, RCC diagnosis is often incidental and is identified through abdominal ultrasound or via computed tomography (CT) scan performed for other medical purposes [22]. However, due to its limitations in specificity and accuracy, CT scan and magnetic resonance imaging (MRI) technologies represent the gold standard for renal mass identification [21]. Percutaneous renal biopsy, on the other hand, is often not considered a reliable diagnostic tool because of RCC heterogenicity and due to the invasiveness of the procedure with a nonnegligible risk of infections and bleeding after biopsy [23]. Recently, confocal microscopy has been proposed as an additive platform for RCC diagnosis and has been tested in both RCC and prostate cancer settings [24,25]. Future trials will reveal the clinical impact of this technique.

## 5. Progression and Course of Disease

Unfortunately, due to its asymptomatic presentation and to the absence of a predictive, reliable molecular biomarkers, in most patients, RCC remains clinically silent until it reaches the advanced stage when the rate of survival decreases dramatically [26]. Approximately 35% of RCCs eventually become metastatic (mRCC), and the most frequent sites of hematological dissemination are the lung, liver, bone, brain, and lymph nodes [27]. However, the biological mechanisms underlying the metastatic process in RCC have not yet been well elucidated and established. Some studies have focused on the circulating tumor-derived extracellular vesicles (EVs), which are shed from the primary tumor and scatter throughout the blood stream. Different types of microRNAs contained within these EVs, as well as cancer stem cells, can promote angiogenesis, thereby facilitating metastatic spread of the disease [28,29]. Other studies focused on investigating the role of the immune system, the negative effects on antitumor immunity (e.g., myeloid-derived suppressor cells, MDSC), and its impact of RCC metastasis. Moreover, other molecular mediators of mRCC have been elucidated in the last years, such as CUB-domain-containing MUC1, a membrane-bound glycoprotein, and the chemokine CXCR4 [30]. All these molecules promote the migration of metastatic cancer cells through HIF-dependent pathways in the setting of VHL [31].

Most recurrences (~85%) are observed within the first three years after radical nephrectomy (RN) or nephron-sparing surgery (NSS), with a mean interval of 29.5 months for patients with stage T2 RCC and 22 months for those with stage T3 [32]. Presently, the prognosis of mRCC remains poor, with a 5-year survival rate of only 12%. In fact, due to the high heterogeneity of RCC at the molecular, genomic, histopathological, and clinical levels, the clinical management of mRCC remains extremely complex and with poor prognosis [33,34]. Several articles have highlighted that although renal histology (ccRCC, pRCC, chRCC, etc.) is routinely applied in clinical practice to define a prognostic scenario for metastatic patients, the biology of tumors may differ across metastatic sites [35]. Notably, Gerlinger and colleagues pointed out that the modifications in the mTOR pathway were variable across sites of metastasis, as well as for SETD2, PTEN, and KDM5C molecular status [36].

## 6. Surgical Management

Despite advances in the understanding of RCC biology, surgery remains the mainstay of curative treatment [37]. Although radical nephrectomy (RN) was historically the standard of care for the management of renal tumors, the early detection of small renal lesions, together with accumulating evidence on the negative effect of RN on renal function over time (e.g., chronic kidney disease (CKD), have led to a more conservative approach [38,39]. Nephron-sparing surgery (NSS), active surveillance, and minimally invasive techniques have been introduced into daily clinical practice with several benefits, thus avoiding surgical over-treatment and the onset of surgical CKD [40,41,42]. The prognosis of RCC is closely related to the aggressiveness at the time of diagnosis. Evidence from several studies has highlighted that cytoreductive nephrectomy plays a crucial role, especially in patients with good performance status and a low volume of metastatic disease [43,44]. The use of metastatectomy and of other treatments such as whole-brain radiotherapy (RT), conventional radiotherapy, stereotactic radiosurgery, stereotactic body radiotherapy, cyberknife RT, and hypofractionated RT represent other medical strategies for the management of mRCC patients [45,46,47].

## 7. Pharmacological Management

The correct time to begin systemic therapy is not yet well established, especially in patients with limited tumor burden and lack of symptoms [48]. Several significant changes have taken place in the oncological management of mRCC. Prior to 2005, the use of cytokines such as interferon and interleukin-2 was adopted as standard medical treatment [49]. Later, both EGF inhibitors and tyrosine kinase inhibitors (TKIs) became leaders in the standard of care for advanced mRCC [50], and most recently, immune checkpoint inhibitors (ICIs), with or without TKIs, have initiated a new era in cancer treatment, offering novel and effective therapeutic approaches for mRCC [51,52,53]. Thanks to the development of new immunological agents for the management of mRCC, studies have shown great interest in evaluating PD1 ligand (PD-L1) expression at the metastatic site [54]. The programmed cell death-1 (PD-1)/PD-L1 pathway represents a crucial checkpoint for the modulation of T-cell-mediated immune responses [55] is responsible for the reversible inhibition of T-cell activity and proliferation, which inevitably leads to anergy. Distinct types of aggressive cancers utilize this escape strategy to downregulate the immune system, avoiding T-cell defense responses. Furthermore, inhibition of the PD-1/PD-L1 promoted by new drugs increases the anticancer immune activity, avoiding the breakout of tumor cells from the host T-cell controls [56].

Nevertheless, both immunotherapy and TKIs can be associated with therapy resistance due to a highly dynamic, adaptive, and heterogeneous tumor microenvironment [57]. For such reasons, one of the main problems mRCC patients encounter during medical therapy is the follow-up of the metastatic burden, which is driven by radiological exams able to detect the progression or regression of the disease. Unfortunately, even in the era of precision medicine, there is a dearth of accurate and tailored markers for mRCC to monitor or predict, through a simple liquid biopsy, its behavior during systemic treatment [58].

## 8. Effects of RCC on Kidney Function

Compromised renal function is recognized as a critical issue in renal cancer patients that can negatively affect kidney and overall survival [59]. Renal cancer patients, in fact, are at increased risk of developing acute and chronic reduction of renal function due to preexisting kidney damage, de novo renal insults during renal surgery and the postoperative period. Lastly, a variety of other medications and certain diagnostic procedures may hasten the progression of renal disease [39].

Chronic kidney disease has been reported in about 25% of kidney cancer patients prior to surgery or anticancer treatments. An analysis of risk factors of both conditions highlighted the role of hypertension, diabetes, older age, male gender, obesity, tobacco abuse, and cystic disease [2,60]. The mentioned risks factors expose kidney cancer patients to an increased risk of developing intra- and postsurgical AKI, as well as CKD in the long-term. Our group recently reported that 64% of 144 patients treated with radical nephrectomy in a tertiary institution for renal cancer developed AKI during the in-hospital stay [38]. Among the same group, 85% of AKI patients developed worsening of renal function at 1 year and fell into more advanced G categories of the KDIGO classification [38]. Interestingly, patients with higher baseline renal function (eGFR > 60 mL/min/1.73 m^2^) developed a greater drop in 1-year eGFR compared to their CKD counterparts (29 vs. 11 mL/min/1.73 m^2^) [38]. Although CKD progression after nephrectomy is related to the degree of histological impairment of nonneoplastic renal tissue, the clinician’s attention should be focused on attenuating the impact of the modifiable risk factors. The ultimate goal, especially in patients affected by more advanced disease, (i.e., metastatic disease) is to slow the rate of renal function impairment and avoid the use of agents or procedures that could worsen renal function.

To minimize loss of renal function prior to surgery, the clinician’s evaluation should start with a correct and thorough assessment of renal function, the management of comorbidities (i.e., optimization of blood pressure and glycemia levels), and the discontinuation of nephrotoxic drugs. Furthermore, the analysis of nonneoplastic renal tissues can provide valuable information about the degree of renal damage and about the presence of paraneoplastic renal disease. The reevaluation of renal function after surgery allows the physician to recommend the most appropriate follow-up procedures. One of the most key areas in the onco-nephrology field regards the use of contrast medium (CM) in patients with reduced renal function. In fact, it has been estimated that more than 10% of all AKI are the result of the administration of iodinated contrast medium for CT scans. These events, defined as postcontrast AKI (PC-AKI) are often the effects of a cumulative burden of nephrotoxic insults that include heart failure; volume depletion; uncontrolled diabetes; anemia; and administration of nephrotoxic drugs including antibiotics, analgesics, and bisphosphonates [61]. Other drugs, such as metformin, show increased toxicity in patients with reduced eGFR and should be withheld at the time of CM administration if eGFR < 30 mL/min/1.73 m^2^ [62]. Importantly, administration of anticancer agents in close temporal proximity to CM administration might significantly increase the likelihood of renal injury. Particularly, cisplatin-treated patients develop a more than 2.5-fold risk of PC-AKI when the CM exposure precedes the chemotherapy cycle by less than 1 week [63]. Other antineoplastic drugs, including targeted agents and ICIs, are known to cause glomerular and tubular injury as well as electrolyte disorders [64]. Their role in the development of PC-AKI is still being investigated, but the damage they cause appears quite evident. The preventive measures of PC-AKI involve supplementing hydration, usually with saline solution, and the choice of iso-osmolar contrast medium, especially in high-risk patients [61]. Despite all the preventive measures, a substantial number of CKD patients still develop PC-AKI in various clinical scenarios [65]. This highlights the need for widely available alternative measures to monitor both clinical responses to anticancer drugs and the early recognition of relapse or metastasis of the renal cancer.

Another critical issue involving renal cancer patients regards the development of renal paraneoplastic syndromes that can be observed after the analysis of nonneoplastic renal parenchyma. Unfortunately, in more than 60% of renal cancer patients, the presence of medical nephropathy is not reported or recognized at the time of tumor diagnosis [66]. Once performed, pathological analysis often reveals the presence of diabetic nephropathy in more than 30% of cases, focal segmental glomerulosclerosis, and hypertensive nephrosclerosis, although other patterns are less frequently reported [66,67].

## 9. Effects of Chemotherapy for RCC on Kidney Function

The development of proteinuric kidney disease is also a consequence of several anticancer agents including tyrosine kinase inhibitors and ICIs currently used in the treatment of metastatic kidney cancer.

Agents targeting VEGF and its cognate receptors (i.e., VEGFR-1, -2, -3) can induce glomerular functional and structural changes and alterations in glomerular repair processes, leading to proteinuria [68]. Although no guidelines are available for the treatment of proteinuria in these patients, careful monitoring before each therapeutic cycle is essential; if 24 h urinary protein exceeds 3 g, treatment withdrawal is recommended.

Hypertension can complicate up to 40% of treatments against VEGF or VEGFR [69] and may depend on reduced cell renewal and lower production of vasodilators such as nitric oxide and prostacyclin, thus leading to vasoconstriction, increased peripheral resistance, and decreased renal excretion of sodium [70]. Although new-onset or worsening of preexisting hypertension in these patients can be considered a marker of treatment efficacy, a strict and personalized medical nephrological or cardiological follow up represents the first strategy to avoid uncontrolled blood pressure levels. The use of anti-hypertensive drugs such as calcium antagonists, RAAS inhibitors or diuretics remains a second-line choice due to the possible side effects of these therapies, such as volume depletion, tachycardia, acute kidney injury or angioedema [71].

Thrombotic microangiopathy is a rare but serious complication of targeted agents, especially those directed against VEGF and VEGFR. Clinical features usually include new-onset proteinuria and hypertension, worsening renal function, anemia, and thrombocytopenia; schistocytes are rarely described. Once these agents are discontinued, renal function usually improves, and proteinuria generally resolves uneventfully [72].

In addition to these chemotherapy-induced renal complications, a wide range of other renal lesions have been described in association with targeted agents, including electrolyte disorders, AKI (due to indirect effects), and worsening of CKD; we recommend referring to specific publications for an extensive evaluation of current, targeted anticancer drugs.

Immune checkpoint inhibitors are a recent class of anticancer drugs that enhance the adaptive immune response by blocking the inhibitory binding between specific T-cell receptors (PD-1) and cytotoxic T lymphocyte-associated antigen-4 (CTLA-4) and their ligands, which are overexpressed in cancer cells [73]. The beneficial antitumor effects can be associated with the loss of self-tolerance, which may cause a wide spectrum of immune-related adverse effects. Renal adverse effects include AKI, mainly due to acute tubulointerstitial nephritis that may occur even up to 18 months after exposure to these drugs [74]. Prompt recognition of renal injury and initiation of steroid therapy may prevent progression to tubular fibrosis [74,75].

Taken together, these conditions raise important clinical issues. First, the development and utilization of liquid biopsies in the follow-up of renal cancer is warranted to limit the burden of PC-AKI. Second, validation of these markers should consider the retention of several solutes, including proteins, having a wide range of molecular weight, that occurs with the reduction of renal function. Lastly, the presence of medical nephropathies including proteinuric disorders may alter the urinary biomarker concentration thereby leading to critical issues in terms of standardization and normalization of the results.

## 10. Circulating Biomarkers in Metastatic RCC

**Figure 1:**
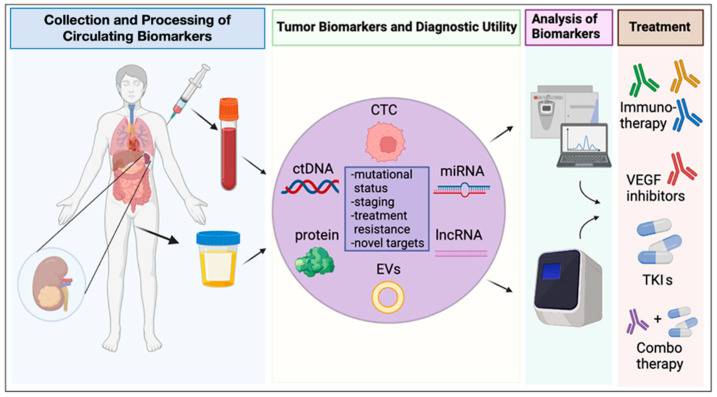
Circulating Biomarkers. Circulating biomarkers can be obtained from biological fluids such as blood and urine. These include circulating tumor cells (CTC), cell-free DNA (cfDNA), circulating tumor DNA (ctDNA), microRNA (miRNA), long-noncoding RNA (lncRNA), circulating proteins, and extracellular vesicles (EVs) (lipids complexed with DNA, RNA, or protein). Matching biomarker profiles with stage- and treatment-resistance phenotypes may allow tailored therapy with immunotherapy, vascular endothelial growth factor (VEGF) inhibitors, tyrosine kinase inhibitors (TKIs), or combinations thereof.

The use of circulating tumor biomarkers has numerous potential advantages over conventional biopsies (Figure 1). Circulating biomarkers are minimally invasive, making them a safer and easier tool, without the risk of bleeding and infections associated with conventional tissue biopsies. In contrast to conventional tumor biopsies of RCC, which are subject to sampling bias due to the high tumor heterogeneity of RCC, circulating biomarkers obtained from the plasma and urine of patients, in a study by Smith et al., captured 90% of the tumor mutations detected in 10 spatially distinct biopsies following nephrectomy [76]. In other words, plasma and urine circulating biomarkers provided a higher fidelity characterization of tumor heterogeneity compared to conventional tissue biopsies. Additionally, the use of circulating biomarkers does not need hospitalization; it allow faster sampling and sequential analysis of a tumors molecular profile over time and during treatment; and it significantly reduces costs from diagnosis to follow-up. The benefits that circulating biomarkers offer over traditional tissue-biopsies are magnified in the case of metastatic tumors such as mRCC, where secondary sites are not clearly detectable or are not easily accessible [77]. There are many promising tumor circulating biomarkers, including nucleic acid-based tumor markers (i.e., circulating tumor DNA and noncoding RNA), proteins, EVs, and tumor cells. These circulating biomarkers and their potential use as predictive biomarkers of response to therapy in mRCC will be discussed in more detail below (Figure 1).

### 10.1. Circulating Nucleic Acids

#### 10.1.1. Circulating Tumor DNA

As tumors grow in size, their constituent cells quickly crowd each other out as they vie for blood supply and nutrients, leaving numerous cancer cells to die, in turn liberating their cell contents into the surrounding extracellular milieu. One of these intracellular constituents is tumor DNA. Successful measurement of circulating tumor DNA (ctDNA), obtained via liquid biopsies, requires for the ctDNA to be present in sufficient quantities and to be readily distinguished from nontumor cell DNA. Thus, detection methods must be sensitive, and the mutational state of the ctDNA must be understood. Indeed, while numerous studies have shown that quantifiably higher levels of intact and fragmented cell-free DNA (cfDNA) can be detected in patients with advanced or mRCC, ctDNA emanating from the RCC is less abundant and more difficult to measure [78,79,80,81,82].

In the case of ctDNA, however, results have been more mixed, largely because of detection and low abundance of ctDNA [76,83,84,85]. Although there are notable exceptions in the literature, the detection appears to be more robust with larger tumors and in metastatic disease [76,85,86,87]. So far, several platforms to assess ctDNA are commercially available, and various clinical studies utilize hotspot panels that sequence specific genes of interest and more recently also allow noninvasive diagnostic and prognostic tools to detect minimal residual disease [88,89]. Usually, a routine blood draw allows the isolation of enough ctDNA, which undergoes library preparation for next-generation sequencing (NGS). This methodology provides higher resolution analytical analysis of single-nucleotide variants (SNVs), fusions, copy number alterations (CNAs), and indels that can guide clinical decision making [77].

The role of ctDNA to predict and monitor treatment response in kidney cancer has also been evaluated [90]. In a recent study, genomic alterations in ctDNA were evaluated in 220 patients with metastatic RCC. At least one genomic alteration was detected in 78.6% of patients, and the most frequent included TP53, VHL, EGFR, NF1, and ARID1A [87]. Among this cohort of patients, 99 patients received first- (sunitinib or pazopanib) or second/later line (nivolumab, everolimus, axitinib, or cabozantinib) treatment, and the median number of ctDNA alterations detected was one. The highest disparity in genomic alterations frequencies in postfirst-line versus first-line was in TP53 (49% vs. 24%), VHL (29% vs. 18%), NF1 (20% vs. 3%), EGFR (15% vs. 8%), and PIK3CA (17% vs. 8%) [87]. Restricting the analysis to later lines versus first-line with vascular endothelial growth factor inhibitors, these differences were even more prominent, particularly for TP53 (64% vs. 31%) and NF1 (29% vs. 4%) [87]. CtDNA has been proposed as a potential tool for obtaining real-time genomic data in metastatic RCC as well as other hematologic malignancies and solid tumors, monitoring the disease progression and allowing for more informed therapeutic decisions. Since the tumor heterogeneity is difficult to address with biopsies, the assessment of the mutational profile in ctDNA might be used to follow the clonal evolution and help a real-time selection of best treatment approaches to offer to patients.

Monitoring of plasma cell-free DNA (cfDNA) levels during a treatment can be a useful marker to predict good or poor response and to monitor patients during follow-up. Plasma cfDNA levels were quantified before treatment and after 4, 8, 12, 16, and 4 weeks in 18 metastatic cRCC patients receiving sorafenib [91]. A significantly lower plasma cfDNA level, measured from 8 weeks to 24 weeks, was found in patients with remission or stable disease compared to those with progression, indicating that higher cfDNA levels could be associated to a poorer outcome. For predicting progression, a sensitivity of 66.7% was achieved at 100% specificity using cfDNA levels at an early stage (8 weeks) [91]. Similarly, the presence of detectable ctDNA was associated with a weaker effect in response to tyrosine kinase inhibitors [80]; the variant allele fraction for seven variants was markedly reduced in subsequent cfDNA samples at the time the partial response was achieved [92]. In a separate study, longitudinal sampling revealed that ctDNA can track disease course and may preempt radiological identification of minimal residual disease or disease progression on systemic therapy [76].

#### 10.1.2. Circulating Noncoding RNA

Along with ctDNA, circulating noncoding RNAs (ncRNAs), including microRNAs (miRNAs), long noncoding RNAs (lncRNAs), and YRNAs, can be passively released by tissue or cell damage or actively secreted as cell-free circulating RNAs carried by EVs, such as exosomes and microvesicles, or bound to lipoproteins [93]. In contrast to ctDNA, RNA is more labile in the blood, particularly coding RNAs such as messenger RNAs (mRNAs) [94], whereas ncRNAs are often complexed with proteins or contained in EVs, which extend their serum half-life and make measurement and quantification/analysis more feasible [94]. Circulating ncRNAs appear to be well-accessible biomarkers in numerous cancer types, including urogenital malignancies, such as kidney cancer [21]. As such, ncRNAs are more frequently studied in the context of liquid biopsies. Relevant studies and the merits of using RNA obtained by liquid biopsies to assess RCC and mRCC are discussed in more detail below.

Several miRNAs have emerged from studies in RCC and mRCC. Analysis of serum samples from 68 patients with ccRCC revealed elevated serum levels of miR-210 compared to normal patient controls along with progressively diminishing levels of miR-210 in ccRCC patients in the weeks following surgical resection of their tumors [95]. In addition, the levels of miR-210 were higher in serum samples compared to patient matched conventional biopsies from normal, nontumor renal parenchyma. A similar result was obtained when examining miR-210 levels in the urine, another form of liquid biopsy, in patients with ccRCC [96,97]. While the levels of some of these microRNAs may serve as signatures for ccRCC, few studies have been undertaken in the context of mRCC with these biomarkers. Others have found numerous miRNA biomarkers that are differentially expressed in mRCC tissue versus RCC tissue (including miR-215), but this has yet to be validated by liquid biopsy [98].

As noted, RNA often complexes with proteins, EVs, and exosomes. Both miRNAs and lncRNAs can be detected when measuring EVs and exosomes in ccRCC and mRCC with urine and serum liquid biopsies [99,100,101,102].

In addition to generalized expression changes in the context of RCC and mRCC, miRNAs and lncRNAs have been demonstrated to medicate acquisition of resistance to treatment regimens in RCC. For instance, in a prospective observational multicenter study including 38 metastatic RCC patients receiving first-line treatment with sunitinib, predictive models were developed on the basis of miRNA expression seen with treatment resistance [103].

Expression patterns of miRNAs can vary with disease progression. For example, it was observed that a poor response group (progression earlier than 6 months after therapy initiation) had alterations in the expression of miR-192, miR-193-3p, and miR-501-3p, whereas a prolonged response group (progression after 18 months from therapy initiation), had alterations in expression of miR-miR-410, miR-1181, and miR-424 [103].

MiR-192 has tumor-suppressive functions in RCC by inhibiting cell migration, invasion, and epithelial-to-mesenchymal transition through targeting ZEB2, MDM2, and TYMS [104]; miR-193-3p functions as a tumor-promoting microRNA by directly targeting PTEN in RCC [105]; miR-501-3p induces G1 phase arrest in RCC cells by targeting the Wilms’ tumor 1-associating protein (WTAP)–CDK2 axis [106]. The level of miR-31-5p carrying EVs in the blood have been analyzed in 40 metastatic RCC patients who experienced progressive disease during sorafenib therapy [107]. The levels of miR-31-5p within EVs were significantly upregulated in sorafenib-resistant disease when compared to those in pretherapy status, suggesting that the increase of miR-31-5p might predict the emergence of resistance [107]. MiR-31-5p within EVs downregulate MutL homolog 1 (MLH1) expression, promoting sorafenib resistance. Indeed, low MLH1 expression was observed in sorafenib-resistant RCC cells, and MLH1 upregulation restores the sensitivity of resistant cell lines to sorafenib. EVs-shuttled miR-31-5p can transfer sorafenib resistance to sensitive cells by directly targeting MLH1 and thus magnify the drug resistance information to the whole tumor [107]. To determine possible secreted biomarkers predictive of response to the combined therapy with vorinostat and bevacizumab, serum levels of miRNAs were investigated pre- and posttreatment [108]. Selected miRNAs differentially expressed between therapy responders and nonresponders subgroups were identified, and miR-605 was found at higher levels in responders compared to nonresponders. MiR-605 acts to interrupt the interaction between p53 and Mdm2 to create a positive feedback loop aiding rapid accumulation of p53 to facilitate its function in response to stress by mediating for instance cellular repair or promoting apoptosis in cells with an overabundance of molecular derangements [109]. As discussed above, urine has been used as a source for liquid biopsies for prognostic and predictive purposes in metastatic RCC [110]. As documented in other studies, miR-210-3p expression was found to be upregulated in both tumor tissues and in urine samples of 21 ccRCC patients, whereas miR-210-3p was significantly reduced in urine samples from disease-free patients from 3 to 12 months, compared to the baseline levels observed at the time of surgery. Finally, in a small subgroup of patients presenting metastatic progression, the urine levels of miR-210-3p correlated with responsiveness to the therapy with sunitinib alone or in combination with pazopanib [110]. MiR-210-3p mediates multidrug resistance of RCC cells via binding with ABCC1 and its subsequent inhibition. Downregulation of miR-210-3p increases ABCC1 expression, thereby enhancing multidrug resistance of RCC cells [111]. Finally, like miRNAs, the ncRNA lncARSR is elevated in plasma from RCC patients and has been implicated in the developing of sunitinib resistance [101]. In addition, similar to miR-210, the levels of lncARSR decreased after tumor resection and were elevated again upon tumor relapse, linking its production to disease progression. Remarkably, the average level of lncARSR in pretherapy plasma was higher in patients with progressive disease during sunitinib therapy than those lacking it and could be correlated with a reduced progression-free survival, even more significantly in the metastatic setting. High lncARSR levels in pretherapy plasma correlated with poor sunitinib response in RCC patients, suggesting that it might predict the emergence of resistance [101]. LncARSR was found highly expressed in primary renal tumor-initiating cells and to promote their self-renewal capacity, tumorigenicity, and metastasis. Mechanistically, lncARSR interacts with Yes-associated protein (YAP) to block its phosphorylation by LATS1, thus facilitating YAP nuclear translocation, where it serves as a transcription coactivator and reciprocally enhanced lncARSR transcription, thus forming a feed-forward circuit [112].

### 10.2. Circulating Proteins

Among the array of biomarkers discussed here, circulating proteins stand out for a number of reasons including measurement techniques, abundance in the blood, and historical experience with markers for RCC (i.e., KIM1) [113]. Some of these same issues serve as drawbacks, however. Plasma proteins are, in general, abundant, making it challenging to isolate proteins that can serve as tumor biomarkers. Nevertheless, a number of promising protein biomarkers have been described that can be detected in the blood of patients with RCC, ccRCC, and mRCC. These include proteins such as KIM1, HIG3, CAIX, IMP3, CD27, CD70, and TRAIL and, in some cases, may correlate with poor survival or metastasis, offering insight into disease progression [114,115,116,117,118,119,120,121,122].

Given the ease of its detection, serum levels of circulating cytokines and angiogenic factors (CAFs) are the most studied biomarkers to predict outcomes of VEGFR and mTOR inhibitor targeted therapies (with antiangiogenic agents such as sunitinib, sorafenib, bevacizumab, pazopanib, axitinib, cabozantinib, temsirolimus, and everolimus). Considering the pharmacological effect and biological activity of these agents, measuring the plasma levels of, e.g., circulating VEGF pathway proteins such as VEGF family ligands (including PlGF, a specific ligand of VEGFR-1), PDGF, and the soluble form of the VEGF receptors (sVEGFR-1, sVEGFR-2, and sVEGFR-3), could be a good strategy to find predictive biomarkers. There are several recent studies demonstrating the potential of tailoring targeted therapy to RCC in accordance with circulating biomarkers. These relevant studies and their major findings are discussed in more detail below.

In a phase II study, Deprimo et al. showed that plasma levels of VEGF, sVEGFR-2, sVEGFR-3, and PlGF could be potential biomarkers of sunitinib pharmacological and clinical effect in 63 patients with metastatic RCC after failure of first-line cytokine-based therapy (IFN-α, IL-2) [123]. Indeed, VEGF and PlGF plasma concentrations increased in many patients after treatment with sunitinib; in contrast, sVEGFR-2 and sVEGFR-3 plasma concentrations were decreased in most patients [123]. These levels were restored to near baseline after 2 weeks off treatment, indicating that these effects were dependent on drug exposure [123]. In addition, significantly larger proportional changes in VEGF, sVEGFR-2, and sVEGFR-3 levels were observed in patients exhibiting objective tumor response compared with those exhibiting stable disease or progressive disease [123].

Rini et al., in another prospective phase II study, also showed that sunitinib modulates plasma soluble proteins VEGF-A, VEGF-C, sVEGFR-3, and PlGF in a cohort of sixty-one patients with bevacizumab-refractory mRCC [124]. In agreement with the study of Deprimo et al. [123], the authors showed that plasma VEGF-A and PlGF levels significantly increased with sunitinib treatment and return to near-baseline levels at the end of the off-treatment periods, while plasma sVEGFR-3 and, to a lesser extent, VEGF-C levels significantly decreased with sunitinib treatment [124]. In addition, lower baseline levels of sVEGFR-3 and VEGF-C were associated with longer progression-free survival (PFS) and objective response rate (ORR), showing potential as predictive biomarkers of response to sunitinib [124]. It was not clear whether the association of plasma VEGF-C and VEGFR-3 levels with sunitinib response is indicative of a subset of patients who are intrinsically less responsive to sunitinib or is specific to the bevacizumab-refractory population and reflective of a bevacizumab resistance mechanism [124]. The lack of correlation between VEGF-A or PlGF levels and PFS could be since the study was based on bevacizumab-refractory patients [124].

Plasma proangiogenic markers were also evaluated by Kontovinis et al. in 42 patients with ccRCC treated with sunitinib [125]. In this study, they showed that plasma sVEGFR2, PDGF, and VEGF-A levels fluctuated during the on–off treatment periods in an analogous way, as has been reported in the other studies [108,123,124]. While sVEGFR2 and PDGF did not show any predictive value, plasma VEGF-A levels were higher in patients that had disease progression than in patients who obtained a clinical benefit [125]. These results were in contrast with what was observed by DePrimo et al. in mRCC patients treated with sunitinib after failure of first-line cytokine therapy [123]. On the other hand, they agreed with the findings of Porta et al., who found that increased baseline serum VEGF-A levels were significantly associated with shorter PFS in a cohort of 85 mRCC patients treated with sunitinib compared with patients with low serum VEGF-A levels [126]. Kontovinis et al. also showed that VEGF-A did not increase in patients who originally obtained a clinical benefit and later progressed while on treatment, implying a different mechanism between primary (disease refractory to treatment) and secondary resistance [125]. This finding was further confirmed in two other studies [127,128].

In a multicenter, prospective, open-label phase II trial (PREINSUT) where sunitinib was used prior to planned nephrectomy in 32 mRCC patients, Mauge et al. found that baseline high levels of VEGF-A, SDF-1, and sVEGFR1, possibly reflecting hypoxia, and low levels of sVEGFR2 were associated with a shorter PFS, while high levels of SDF-1 and sVEGFR1 were associated with shorter overall survival (OS); during sunitinib treatment, SDF-1 and PDGF-BB were associated with primary renal tumor (PRT) response, sVEGFR2 with PFS, and SDF-1 and sVEGFR1 with OS [127].

In another work, a phase III trial of sunitinib versus interferon-alpha (IFN-α) in mRCC, Harmon et al. showed in a subset of 60 patients (33 treated with sunitinib vs. 30 treated with IFN-α) that baseline VEGF-A and IL-8 may have prognostic value, with high plasma levels being unfavorable. On the other hand, low baseline levels of plasma sVEGFR-3 were significantly associated with improved response to sunitinib [128]. Plasma levels of VEGF were assessed as predictive biomarker not only for sunitinib treatment but also for other antiangiogenic agents.

The relationship between baseline plasma levels of VEGF and sorafenib benefit was assessed in a phase III multicenter, randomized, double-blind, placebo-controlled study of treatment with sorafenib in 712 ccRCC patients with unresectable and/or metastatic tumor who experienced treatment failure with one prior systemic therapy (Treatment Approaches in Renal Cancer Global Evaluation Trial, TARGET). This study suggested that patients with levels above the 75th percentile at baseline may benefit more from sorafenib (with respect to PFS) than those with low levels, although sorafenib benefit was apparent in both groups [129]. In the same TARGET trial, on the other hand, Peña et al. showed that baseline plasma levels and changes during treatment in plasma levels of sVEGFR-2, CAIX, TIMP-1, and Ras p21 were not associated with sorafenib benefit [130].

In another multicenter, single-arm phase I/II clinical trial of the HDAC inhibitor vorinostat and the VEGF blocker bevacizumab in metastatic ccRCC patients previously treated with different drugs (i.e., sunitinib, sorafenib, axitinib, interleukin-2, interferon, and temsirolimus), the authors evaluated serum levels of secreted growth factors (VEGF, FGF2, and HGF), invasion and metastatic markers (SDF and OPN), and cytokines (IL-8) [108]. VEGF decreased significantly in serum but not in plasma of all the patients following treatment, making it difficult to associate biological and clinical outcome. However, the decrease in serum VEGF levels did not correlate with response [108]. In addition, FGF2, SDF, OPN, and IL-8 decreased significantly in the group of patients who achieved an objective response as compared with the patients who had progressive disease, suggesting that modulation of chemokines may be associated with response to treatments targeting the HIF/VEGF axis [108].

Correlative studies identified potential predictive and pharmacodynamic biomarkers, including VEGF-A, in a phase Ia multicenter, dose-escalation and dose-expansion trial of atezolizumab (NCT01375842) in a cohort of 70 patients with mRCC (many of which had received previous systemic therapies) [131]. In this study, plasma VEGF-A decreased in responders but was stable in patients with stable disease or progressive disease, suggesting that it could be a noninvasive tool to identify on-treatment markers of response to atezolizumab monotherapy [131]. In addition, on-treatment decreases in acute-phase proteins, including ferritin, complement C3, vitamin D–binding protein, and macrophage inflammatory protein-1a, were significantly associated with longer OS, suggesting that patients with decreased systemic inflammation derive greater OS benefit. These inflammatory markers could additionally provide early on-treatment biomarkers of atezolizumab response. Lower baseline levels of multiple acute-phase proteins, including von Willebrand factor, serum amyloid P component, a-1-antitrypsin, and fibrinogen, were also associated with longer OS [131]. In another study, high levels of IL8 in plasma, PBMCs, and tumors were associated with decreased efficacy of atezolizumab [132].

In a randomized phase III trial of cabozantinib vs. everolimus in advanced RCC (METEOR), VEGF, sVEGFR2, IL-8, CA9, HGF, MET, GAS6, and AXL were assessed as biomarkers in plasma of 621 randomized patients [133]. In univariate analyses, patients with low levels of HGF had a more favorable prognosis for PFS and OS in both treatment arms. In addition, low baseline AXL and VEGF were associated with cabozantinib benefit (in terms of both PFS and OS). Low AXL was also predictive of relative improvement in PFS for cabozantinib versus everolimus [133]). In multivariable analysis, low baseline HGF levels was an independent prognostic factor for improved PFS for both cabozantinib and everolimus; low HGF, GAS6, and VEGF were independent prognostic factors for improved OS with cabozantinib; and no biomarkers were independent prognostic factors for OS with everolimus. Pharmacodynamic changes for cabozantinib targets were consistent with previous reports with cabozantinib treatment, with all biomarkers increasing except for sVEGFR2, which decreased in both arms [133]. Although on-treatment changes in some biomarkers (HGF, VEGF, IL-8) appeared prognostic for improved PFS or OS with cabozantinib, in a univariate analysis, none of these were independent prognostic factors in multivariable analyses [133].

In a randomized study of sorafenib alone or sorafenib + IFN-α, Zurita et al. conducted a CAF profiling analysis in 69 patients with mRCC [134]. On univariate analyses, several CAFs correlated with PFS, but on multivariate analysis, only IL-5, M-CSF, and EGF showed independent prognostic value [134]. In addition, low baseline levels of osteopontin and VEGF predicted superior PFS with sorafenib + IFN-α as compared with sorafenib alone, showing promise as potential biomarkers able to identify groups of patients who experienced different degrees of benefit from sorafenib versus sorafenib + IFN-α [134].

VEGF was also included in one of the composite biomarker scores (CBSs) constructed in an exploratory retrospective analysis of a randomized, phase II, open-label, multicenter trial of lenvatinib, everolimus, or their combination as second-line treatment in patients with mRCC previously treated with VEGF-targeted therapy (NCT01136733) [135]. The five-factor PFS–CBS or OS–CBS included the five biomarkers most strongly associated with PFS (HGF, MIG, IL-18BP, IL-18, ANG-2) or OS (TIMP-1, M-CSF, IL-18BP, ANG-2, VEGF), respectively, among the 40 biomarkers tested [135]. The two-factor CBS included only the biomarkers common to PFS–CBS and OS–CBS (IL-18BP, ANG-2) [135]. Patients in the PFS–CBS-high (five-factor) and OS–CBS-high (five-factor) groups appeared to have improved PFS and OS with lenvatinib + everolimus combination therapy compared with everolimus monotherapy in univariate and multivariate analysis and showed promise as a predictive tool. On the other hand, the two-factor-CBS appear to predict PFS but not OS [135].

In a prospective correlative study, authors analyzed 30 plasma cytokines, including VEGF, at baseline and 1 month after starting treatment as candidate predictive biomarkers in 56 mRCC patients who were planned for treatment with either a vascular endothelial growth factor–tyrosine kinase inhibitor (VEGF–TKI) or immune checkpoint inhibitor (ICI) [136]. Patients with clinical benefit from VEGF–TKIs had significantly lower baseline levels of IL-6, IL-1RA, and granulocyte colony-stimulating factor (G-CSF), as well as significantly lower IL-13 and granulocyte macrophage CSF (GM-CSF) and significantly higher VEGF after 1 month of treatment compared to patients with no clinical benefit. On the other hand, patients with clinical benefit from ICIs had significantly higher levels of interferon-γ (IFN-γ) and IL-12 1-month posttreatment. These data further encourage future in-depth study of plasma cytokines as biomarkers for immune phenotype stratification in order to provide more personalized treatment for metastatic RCC [76].

In similar studies, plasma levels of different interleukins, particularly IL-6 and IL-8, were assessed as predictive biomarker in several studies with different therapeutic approach for mRCC and were associated with different outcomes. Plasma IL-6 was identified and confirmed as a predictive biomarker of response to sunitinib by Pilskog et al. [137]. In fact, in 46 patients with metastatic or nonresectable ccRCC treated with sunitinib, low plasma IL6 at baseline was associated with significant response to sunitinib and improved PFS, suggesting that upregulation of plasma IL-6 might represent an important mechanism of resistance [137]. In addition, they showed that patients with high plasma IL-6 signal transducer levels (IL6ST) at baseline showed significantly improved OS, whereas patients with a decrease in concentration of plasma IL-6 receptor α (IL6Rα) between baseline and 12 weeks posttreatment showed significantly improved PFS [137]. In line with these results, Porta et al. showed that, in a large percentage of advanced RCC patients treated with sunitinib, disease progression is preceded by a significant increase in IL-6 and also of other two proangiogenic cytokines (bFGF and HGF) [138]. In another work, the authors found that high plasma baseline levels of IL-6 and HGF and that of other two cytokines (CXCL11 and CXCL10) are associated with worse outcome in a cohort of 60 mRCC patients treated with sunitinib (*n* = 51), pazopanib (*n* = 4), or both (5) [139].

In addition, in a cohort of 90 patients with mRCC treated with sunitinib, Mizuno et al. found that baseline levels of IL-6, IL-8, and high-sensitivity C-reactive protein (CRP) were significantly higher in patients who progressed when compared to those with clinical benefit [139]. As such, these biomarkers showed promise as predictive biomarkers of response to first-line sunitinib treatment in patients with mRCC [140]. Baseline CRP was confirmed as a significant predictive factor of sunitinib response and a prognostic factor of survival in mRCC patients in another study [141]. In this study, normal CRP at baseline was associated with statistically significant objective response to treatment and was also associated to improved PFS and OS, as assessed in 38 mRCC patients treated with sunitinib [141]. CRP seems the most promising biomarker with prognostic value found in a cohort of 110 patients with mRCC treated with low-dose subcutaneous IL-2 based-immunotherapy, where low/normal CRP correlated with better survival in a univariate and multivariate analysis [142].

In a retrospective analysis of phase II and phase III trials of pazopanib treatment in mRCC patients, Tran et al. have confirmed that baseline plasma concentrations of some cytokine (including IL-6 and IL-8) and angiogenic factors (CAFs) CAF profiles could provide prognostic information beyond that of standard clinical classification and identify markers predictive of pazopanib benefit in patients with metastatic renal cell carcinoma [143]. In this work, the authors used a three-step approach for screening, confirmation, and validation of prospective CAF biomarkers in a total cohort of 559 patients [143]. The results obtained showed that high baseline levels of selected cytokines (IL6, IL8, and osteopontin) were negative prognostic factors in patients with mRCC [143]. They also showed that patients with increased levels of cytokines, especially IL6, had a worse prognosis but greater relative benefit from pazopanib (as compared to placebo) [143]. IL-6 was also useful to identify a subgroup of patients who benefitted from the addition of naptumomab estafenatox (Nap) in a randomized, open-label, multicenter, phase II/III study of naptumomab estafenatox (Nap) + IFNa versus IFNa in 513 mRCC patients [144]. In this study, OS and PFS were similar in patients receiving Nap + IFNa and in patients receiving IFNa alone [144]. However, a subgroup of patients having low plasma baseline concentrations of anti-SEA/E-120 and IL6 benefitted from the addition of Nap in terms of both PFS and OS. Thus, Nap + IFN might improve PFS and OS in a baseline biomarker-defined mRCC patient subgroup [144]. The antitumor effects (prolonged OS) of Nap in this subgroup of patients could be due to induction of T-cell activation and expansion (through IL-2 induction in plasma) [145].

A study conducted by Voss et al. was the first with the aim to identify biomarkers predictive of benefits in patients receiving either VEGFR or mTOR TKIs in the first-line setting. The authors attempted this issue in a randomized phase II trial of sunitinib vs. everolimus (RECORD-3), assessing 121 candidate soluble proteins biomarkers in the plasma of 442 randomized patients to test their association with the therapeutic effects to both agents. These candidate soluble protein biomarkers were associated with angiogenesis, cancer, inflammation, metabolism, tissue remodeling, and kidney damage [146]. They showed that baseline levels of multiple soluble biomarkers correlated with benefit from everolimus and/or sunitinib, independent of clinical risk factors. However, one of the main findings of this work was the identification of a composite biomarker score (CBS) obtained using the five everolimus-specific biomarkers (CSF1, ICAM1, IL-18BP, KIM1, TNFRII) showing the strongest association with therapeutic effect. These biomarkers are functionally involved in inflammation/immune response. These same authors also showed significantly better outcomes for everolimus-treated patients with high compared with low CBS, with 42.5-fold longer median PFS1L (HR, 0.43; *p* < 0.0001). For sunitinib-treated patients, the CBS was not significantly associated with outcome. A similar PFS1L was observed in both treatment arms among the patients with a high CBS score [146].

In another study, a prospective phase II multicenter trials in ccRCC patients initiating sunitinib (54 patients) or bevacizumab (45 patients) in the first-line metastatic setting (SUVEGIL and TORAVA trials), Dufies et al. showed that a cytokine involved in inflammation and angiogenesis, CXCL7, may be considered as a predictive marker of sunitinib efficacy for ccRCC patients. In fact, patients with CXCL7 plasmatic levels above the cut-off (250 ng/mL) at baseline had a significantly longer PFS. These results were confirmed in a retrospective validation cohort of 31 mRCC patients treated with sunitinib [147]. Other candidate biomarkers of response to sunitinib could be serum 20S proteasome, whose levels were lower in patients responding to sunitinib than in patients with stable disease and progressive disease [148] and plasma level of the N-terminal precursor of brain natriuretic peptide (NT-pro-BNP) [149]. Increased plasma NT-pro-BNP strongly correlated with clinical outcomes. In fact, a statistically significant increase in NT-pro-BNP was observed in patients with disease progression when compared to those obtaining a clinical benefit [149].

Soluble form of PD-1 and PD-L1 could be candidate predictive biomarkers of anti-PD-1/PD-L1 immunotherapy response in mccRCC. Incorvaia et at. showed that high baseline plasma levels of sPD-1 and sPD-L1 were associated with a longer PFS to nivolumab treatment in mccRCC [150]. Similarly, for BTN3A1, a protein belonging to the butyrophilin 3A subfamily implicated in cancer immune surveillance. In addition, high sPD-1 and sBTN3A1 levels were also associated with best overall response and objective response rate to nivolumab treatment [150]. Therefore, plasma levels of soluble PD-1, PD-L1, and BTN3A1 can predict response to nivolumab treatment in mccRCC patients. Among these, sPD-1 resulted the strongest predictive biomarker [150]. The levels of sPD-L1 and sPD-1 were also evaluated from plasma samples of mccRCC patients before they received a first-line treatment with VEGFR inhibitor sunitinib (50 patients) or the anti-VEGF bevacizumab (37 patients) [151]. The results indicated that high levels of sPD-1 or sPDL1 were independent prognostic factors of shorter PFS in the sunitinib group, while they were not correlated to PFS under bevacizumab treatment. Thus, mccRCC patients with high baseline plasmatic levels of sPD-L1 or sPD-1 are poor responders to sunitinib [151].

In agreement with these results, Larrinaga et al. demonstrated that sPD-L1 could be a marker of treatment response in metastatic ccRCC patients treated with systemic therapies, mainly TKIs, where low plasma sPD-L1 predict favorable response to treatment [152]. These results were confirmed in an exploratory pilot study that enrolled 20 mRCC patients treated with TKI [153]. Thus, high levels of sPD-L1 are associated with poor response to TKI treatment [153]. The authors also showed that sPD-L2 was significantly downregulated in responsive patients. In addition, low levels of IFN, both at baseline and after 3–4 months after starting treatment, correlated with a better response to TKI therapy. High levels of sCTLA4 were also significantly correlated with failure to response during TKI treatment. It is possible that the increase in sPD-L1 and sCTLA is linked to the high levels of IFN. Indeed, IFN-induced PD-L1/2 expression could be a mechanism of adaptive immune resistance to immune checkpoint (IC) therapy [153].

Most clinical trials reported above included only or predominantly RCC with clear cell histology (ccRCC). This makes it exceedingly difficult to tailor the treatment of metastatic RCC with nonclear cell histology (nccRCC). ccRCC were analyzed together with ccRCC or treated as a unique separate group (even if comprising various histological subtypes that are vastly different) because the individual subtypes are rare. Most of the nccRCCs consist of papillary and chromophobe subtypes. There were three different clinical trials evaluating predictive biomarkers in metastatic nccRCC patients treated with sunitinib or everolimus.

In a phase II study, Bilen et al. evaluated plasma levels of a group of 38 cytokines and angiogenic factors (CAFs) in 53 advanced nccRCC patients treated with sunitinib. High levels at baseline of sTNF-RI, TNF-α, sIL-2Ra, IL-8, IL-9, PDGF-AA, and TGF-α were associated with a low response to sunitinib and could be candidate predictive biomarkers of response to sunitinib to be validated in larger trial [154].

In an international, randomized, prospective clinical trial comparing sunitinib and everolimus (ASPEN, NCT01108445), Armstrong et al. found that, in 99 patients with metastatic nccRCC, several plasma angiokines and immunomodulatory chemokines were associated with poor prognosis during treatment [155]. The most promising one was osteopontin (OPN), whose levels were associated with poor-risk disease, poor PFS and OS, and increased at treatment resistance during therapy [155]. They also identified other consistent candidates: TIMP-1, thrombospondin-2 (TSP-2), HGF, and VCAM-1 were associated with poor-risk disease and poor OS, while SDF-1 was associated with improved survival [155]. In addition, they found that plasma levels of some angiokines increased over time during disease progression: Ang-2, CD-73, HER-3, HGF, IL6, PIGF, PDGF-AA, PDGF-BB, SDF-1, TGF-b1-b2, TGFb-R3, TIMP-1, TSP-2, VCAM-1, VEGF, and VEGF-R1 in patients treated with everolimus, and CD-73, ICAM-1, IL6, PlGF, SDF-1, TGF-b2, TGFb-R3, TIMP-1, TSP-2, VEGF, VEGF-D, and VCAM-1 in patients treated with sunitinib [155].

Finally, in another study, a phase II ESPN trial comparing first-line sunitinib to everolimus in previously untreated patients with nccRCC, Msaouel et al. identified different candidate prognostic and predictive circulating biomarkers [156]. They analyzed pre-treatment concentrations of a set of 30CAFs in plasma from 37 patients treated with everolimus (*n* = 16) or sunitinib (*n* = 21). Their results showed that high levels of soluble glycoprotein 130 (sgp130) were significantly predictive of a longer PFS with sunitinib compared with everolimus [156]. Furthermore, significantly shorter PFS was noted, independently of treatment arm, in patients with high levels of IL-8, IL-13, and soluble tumor necrosis factor receptor II (sTNFRII) [156]. High IL-8 levels were also associated with significantly shorter OS [156].

### 10.3. Circulating Tumor Cells

Circulating tumor cells (CTCs) can be found in liquid biopsies from metastatic RCC patients, potentially providing a better representation of intra- and intertumoral heterogeneity, leading to the development of panels with predictive utility. Usually, CTC isolation from blood is carried out by employing antibodies recognizing tumoral markers. More selective antibodies with higher avidity for tumor antigens or biomarkers is key, however, to overcome intrinsic limitations of this method: low detection rate or high false positives. Moreover, CTCs from RCC patients are particularly heterogeneous, both at phenotypic and genotypic levels, due to the involvement of chromosomal remodeling genes in RCC etiology [157].

Most conventional approaches for capturing CTCs use an EpCAM-based enrichment strategy, but it is limited to cancers displaying low or no EpCAM expression, including RCC. Bade et al. used carbonic anhydrase (CA) IX and XII to identify circulating CTC from 29 patients with metastatic RCC treated with TKI of immunotherapy or a combination of both and evaluated them for the PD-L1 and HLA-I expression [158]. CTC enumeration and expression of PD-L1 and HLA-I correlated with disease progression and treatment response respectively, and the longitudinal assessment of a patients’ subset demonstrated potential for CTC enumeration as a pharmacodynamic biomarker [158]. Supporting data to this flow were from the employment of CD147 together with CAIX, which demonstrated significantly higher efficiency for capturing RCC CTCs in peripheral blood [159].

In another study, the authors investigated the presence of CTC with epithelial, mesenchymal, stem cell-like, or mixed-cell characteristics at different time points during antiangiogenic therapy [160]. The presence and quantity of N-cadherin-positive or CD133-positive CTC were associated with reduced progression-free survival. In addition, an inverse correlation between high expression of HIF1A, VEGFA, VEGFR, and FGFR and the presence of N-cadherin-positive and CD133-positive CTC was shown [160]. Two CTC subpopulations were identified in the TARIBO trial. A total of 21 blood samples were serially collected from 10 patients with metastatic RCC. The results were as follows: epithelial CTC (eCTC) and nonconventional CTC (ncCTC) lacking epithelial and leukocyte markers, with a positivity rate of 28% and 62%, respectively, implied that CTC detection in RCC might be improved by the detection of subclonal populations, making it important to identify potential switches occurring during the treatment and supporting prompt treatment adjustments [161]. Thus, CTCs offer an additional, potentially rich, marker for analysis when assessing the clinical utility of liquid biopsies.

## 11. Conclusions

**Figure 2:**
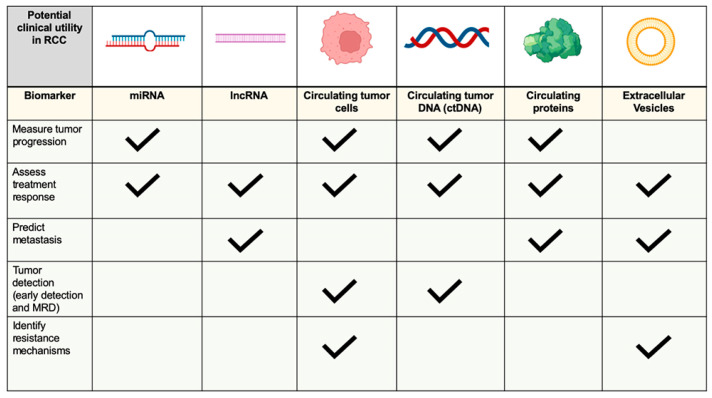
Circulating biomarkers and their potential clinical utility in the oncological management of mRCC patients.

Circulating biomarkers are a unique, rapid, and noninvasive tool to help early detection of cancer, comprehensive genomic profiling, and longitudinal monitoring of cancer cells. Circulating biomarkers have many potential advantages: they can be used to track tumor changes over time, monitor response to therapy, and guide optimal treatment selection (Figure 2). These and other advantages may mean circulating biomarkers replace tissue biopsies in the future. In this intriguing perspective, circulating biomarkers tools could provide in the future a real tailored treatment selecting patients who will really benefit from an oncological therapy. Therefore, the new future medical algorithm related to mRCC patients in clinical practice will comprehend not only radiological techniques but also the routinary use of these circulating biomarkers.

However, this novel and useful tool requires further validation in large-scale cohort studies to determine effectiveness and sensitivity.

## 12. Information Sources and Search Strategy

Bibliographic searches were performed using MEDLINE (via PubMed). The search used both free text and MeSH terms and “English” language filters and was conducted for publications between 2003 and 2021. A manual search of bibliographies in included studies was also performed.

## Data Availability

Not applicable.
